# The evolving concept of liver cancer stem cells

**DOI:** 10.1186/s12943-016-0572-9

**Published:** 2017-01-30

**Authors:** Kouki Nio, Taro Yamashita, Shuichi Kaneko

**Affiliations:** 1 Lineberger Comprehensive Cancer Center, The University of North Carolina at Chapel Hill, Marsico Hall 5229 M, 125 Mason Farm Rd, 27599 Chapel Hill, NC USA; 2 Department of Gastroenterology, Kanazawa University Graduate School of Medical Science, 13-1 Takara-Machi, 920-8641 Kanazawa, Ishikawa Japan

**Keywords:** Liver cancer, Cancer stem cell, Niche, Chemoresistance

## Abstract

Liver cancer is an often fatal malignant tumor with a high recurrence rate and chemoresistance. The major malignant phenotypes of cancer, including recurrence, metastasis, and chemoresistance, are related to the presence of cancer stem cells (CSCs). In the past few decades, CSCs have been identified and characterized in many tumors including liver cancer. Accumulated evidence has revealed many aspects of the biological behavior of liver CSCs and the mechanism of their regulation. Based on these findings, a number of studies have investigated eradication of liver CSCs. This review focuses on the recent advances in our understanding of the biology of liver CSCs and the development of strategies for their treatment.

## Background

Liver cancer is one of the most common cancers worldwide and has a high mortality rate [1, 2]. Among primary liver cancers, hepatocellular carcinoma (HCC) is the major histological subtype and accounts for 70–85% of total liver cancer cases [1]. When diagnosed at an early stage, patients generally undergo surgical resection or liver transplantation according to their hepatic reserve. However, liver cancer is often difficult to treat surgically because many cases are diagnosed at an advanced stage, even at the time of initial diagnosis. Even after surgical treatment, liver cancer recurs frequently and metastasizes. Although chemotherapy, including molecular targeting therapy, is a treatment option for patients with advanced liver cancer, its therapeutic effects are limited, resulting in poor overall survival. The development of cancer recurrence, metastasis, and chemo- and radioresistance in a solid tumor is attributed to the presence of cancer stem cells (CSCs) [3]. In liver cancer, accumulating evidence has demonstrated the existence of a small subset of cancer cells with stem cell properties (self-renewal and differentiation) and several CSC markers have been identified, including CD133, CD90, CD44, oval cell marker OV6, EpCAM, CD13, CD24, DLK1, α2δ1, ICAM-1, CD47, Lgr5, and keratin19 [4–16]. At present, liver CSCs are considered an important targeting subset for the successful treatment of liver cancer. In this review, we summarize the current understanding of the biology of liver CSCs and recent advances in their clinical diagnosis and treatment.

### The hierarchical CSC concept and the origin of liver CSCs

Phenotypic and functional tumor heterogeneity, which is observed in many tumors including liver cancer [17–19], can arise through stochastic genetic [17] or epigenetic [20] changes, or in response to extrinsic environmental differences [21], or from the hierarchical organization of CSCs [22, 23]. In the hierarchical CSC concept, which was first proposed in the 1970s [24], CSCs are present in the biological hierarchy of cancer and have the capacity of self-renewal, multi-lineage potency, and extensive proliferation, resulting in the presence of heterogeneous cells within a tumor. Even though the existence of liver CSCs has been explored by the identification of several surface markers in freshly resected HCC specimens using antibodies and/or flow cytometry-based cell separation methods, their origin remains to be determined [25, 26].

The transformation of liver stem/progenitor cells has been considered one possible origin of liver CSCs. Indeed, CSCs share similar features with normal stem cells, for example, self-renewal and pluripotency, and liver CSCs are identified and classified using normal liver stem/progenitor cell markers, such as EpCAM [27], Lgr5 [28], CD133 [29], and CD24 [30]. Many types of liver cancer develop as a result of a long-lasting inflammation/regeneration process that is induced by chronic viral infection (e.g., hepatitis B virus [HBV]/hepatitis C virus [HCV]), alcohol, or non-alcoholic fatty liver disease. In this process, the expansion of stem/progenitor cells, accumulation of genetic and/or epigenetic changes, and alteration of the microenvironment occur continuously, resulting in the initiation and/or promotion of liver cancer [31]. Furthermore, this process might facilitate the transformation of hepatic stem/progenitor cells into liver CSCs [32–34].

However, liver CSCs do not necessarily originate from only transformed normal stem/progenitor cells, Various cell types, including mature hepatocytes and biliary cells, can be a source of hepatocytes by initializing stem cells during liver regeneration [35]. This initializing process implies another possible origin of CSCs; namely, differentiated cells might be transformed into CSCs due to genetic/epigenetic alteration during cell initialization in the liver injury/regeneration process. Holczbauer et al. investigated the ability of distinct differentiated hepatic lineage cells to acquire CSC properties by stable co-transduction of oncogenic H-Ras/SV40LT into murine hepatic progenitor cells, hepatoblasts, and adult hepatocytes. They found that all transduced hepatic lineage cells can be reprogrammed into CSCs by genetic/epigenetic alterations [36].

Moreover, CSCs may originate from non-CSCs by the activation of “dedifferentiation” [31]. In fact, some evidence of the dedifferentiation of mature cells into CSCs has been accumulated in solid cancer [37, 38]. Recently, Liu et al. reported that the chromatin remodeling factor CHD1L promotes the dedifferentiation of HCC and confers stem cell-like properties on these cells by opening chromatin [39].

**Fig. 1:**
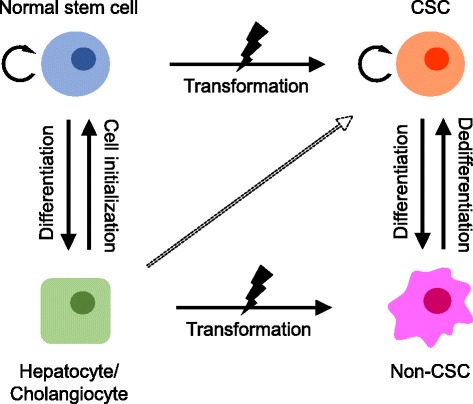
The origin of liver CSC

These findings imply that stem/progenitor cells, mature parenchymal cells, and differentiated liver cancer could be the origin of liver CSCs via “transformation,” “cell initialization,” and “dedifferentiation,” respectively (Fig. 1).

Intrahepatic cholangiocarcinoma (ICC) is the second most frequent histological subtype in liver cancer and is also a treatment refractory malignancy with a high mortality. Originally, ICC has been thought to derive from malignant transformation of mature cholangiocytes composing intrahepatic bile ducts. Interestingly, however, two independent groups demonstrated using cell fate lineage tracing in mice that ICC arises from hepatocytes, rather than cholangiocytes or hepatic stem/progenitors, through Notch/Akt-mediated conversion of hepatocytes into biliary lineage cells [40, 41]. This finding might provide some insights considering “cell reprogramming” related to hepatic stem cell lineage and carcinogenesis.

### Cell surface markers and their function in liver CSCs

Since the first evidence of CSCs in acute myeloid leukemia [42], the existence of CSCs and the identification of CSC cell surface markers have been investigated in many tumors, including liver cancer, by the analysis of immunogenic, tumorigenic, or functional characteristics [23]. Currently, a number of cell surface proteins have been identified as liver CSC markers (Table 1).

**Table 1:** Identified liver CSC markers and their functions

Marker	Author (year)	Function in liver CSCs	Reference
CD133	S. Ma [4]	Regulation of neurotensin/IL-8/CXCL1 signaling	[4, 43]
CD90	Z. Yang [5]	Not reported	[5, 6]
CD44	Z. Yang [6]	Regulation of TGF-beta-mediated EMT, Reduction of ROS through xCT stabilization	[6, 47, 48]
OV6	W. Yang [7]	Not reported	[7]
EpCAM	T. Yamashita [8]	Activation of Wnt signaling, Induction of Wnt-regulated CSC-like gene signature	[8, 52, 53]
CD13	N. Haraguchi [9]	Cell protection from apoptosis via the ROS scavenger pathway	[9]
CD24	T. K. Lee [10]	STAT3-mediated NANOG regulation	[10]
DLK1	X. Xu [11]	Not reported	[11]
α2δ1	W. Zhao [12]	Calcium influx regulation function through L- and N-type voltage-gated calcium channels	[12]
ICAM1	S. Liu [13]	Not reported	[13]
CD47	T. K. Lee [14]	Regulation of CSC properties by cathepsin S/protease-activated receptor 2 paracrine loop	[14]
Lgr5	Z. J. Lei [15]	Not reported	[15]
Keratin19	T. Kawai [16]	Not reported	[16]

### CD133

CD133, which is a primitive marker of hematopoietic stem cells, neuronal stem cells, and liver stem/progenitor cells [29], was identified as a liver CSC marker in 2007 [4]. Despite the fact that CD133-positive cells represent a small population of the total number of cells in human tumor tissue [43], CD133 is clinically significant because patients with high CD133 expression have poor overall survival and higher recurrence rates compared with patients with low CD133 expression [44]. Functionally, CD133 plays a role in the maintenance of CSC properties by regulating neurotensin, interleukin (IL)-8, CXCL1, and MAPK signaling [45].

### CD90

Following the identification of CD133, CD90 was identified and characterized as a marker of liver CSCs. Yang et al. found that CD90+ HCC cells, but not CD90− cells, display tumorigenic and metastatic potential [5, 6]. Both cell line-derived CD90+ cells and liver cancer patient tissue-/blood-derived CD45− CD90+ cells generate tumor nodules in immunodeficient mice, and serial transplantation of xenografts derived from CD90+ cells produces tumor nodules in a second and subsequently third batch of immunodeficient mice [6]. These results also suggested the existence of circulating CSCs in the blood. An obvious role of CD90 in liver CSCs has not been reported.

### CD44

CD44 has been used widely to characterize CSCs in combination with other cell surface markers in several solid tumors. Representatively, a CD44+ CD24−/low cell population was isolated in human breast cancer as the first CSC found in a solid tumor [46]. In terms of HCC, CD44 gives distinct cell features to the CD133+ or CD90+ CSC population. Zhu et al. indicated that CD133+ CD44+ tumor cells possess more stem cell properties, including extensive proliferation, self-renewal, and the ability to give rise to differentiated progeny, and can initiate tumor growth in NOD/SCID mice at very low cell numbers, compared with CD133+ CD44− tumor cells [47]. In addition, Yang et al. showed that CD90+ CD44+ cells demonstrate a more aggressive phenotype than CD90+ CD44− cells and form metastatic lesions in the lung of immunodeficient mice [5]. Two functions of CD44 in CSC maintenance have been reported: CD44 regulates TGFβ-mediated epithelial-mesenchymal transition (EMT) [48]; and a CD44 variant regulates redox status by stabilizing xCT to protect CSCs against reactive oxygen species (ROS) [49].

### EpCAM

EpCAM, a type 1 transmembrane glycoprotein composed of a large N-terminal extracellular domain (EpEx) linked to a short C-terminal fragment (EpICD) by a single-transmembrane domain, has been recognized as one of the most representative and successful markers used in isolating liver stem cells [35]. EpCAM was identified initially as an early biomarker for HCC [50]. It was further classified as a poor prognostic subtype in combination with AFP in HCC [51]. On the basis of transcriptome analysis on a cohort of primary HCC samples, EpCAM+ HCC displayed a distinct molecular signature with features of hepatic progenitor cells, including the presence of known stem/progenitor markers, whereas EpCAM− HCC expressed genes with features of mature hepatocytes [51]. In addition, EpCAM+ HCC showed hepatic cancer stem cell-like traits, including self-renewal and differentiation, and were highly invasive and tumorigenic [8]. We further demonstrated that in comparison with CD90+ HCC, EpCAM+ HCC is highly associated with poorly differentiated morphology, high serum AFP levels, and a low incidence of distant organ metastasis [52]. This classification approach using EpCAM and CD90 might provide for a distinct clinical outcome and therapeutic approach in HCC patients. Mechanistically, EpCAM is one of the Wnt/β-catenin signaling target genes in HCC [53], and the activation of Wnt/β-catenin signaling enriches the EpCAM+ cell population [8]. Simultaneously, EpCAM also activates the Wnt/β-catenin signaling by regulating intramembrane proteolysis (RIP)-mediated EpICD release [54]. Most recently, Mani et al. reported that EpCAM-regulated RIP induces the activation of canonical Wnt signaling as well as the expression of a Wnt-regulated CSC-like gene signature in the presence of HBV infection [55]. These data suggest that EpCAM is strongly related to the maintenance of stem cell properties through the activation of Wnt/β-catenin signaling.

### Other markers

OV6, which was originally classified as a marker of hepatic progenitor cells, was also identified in a subpopulation of cells with a high ability to form tumors in vivo and with substantial resistance to standard chemotherapy [7]. OV6+ cells also exhibited strong invasive and metastatic potential both in vitro and in vivo [56]. CD13 was identified as a novel cell surface marker for CSCs by Haraguchi et al.[9]. They found that CD13+ HCC cells were CSCs enriched in a side population of cells from several HCC cell lines, predominated in the G0 phase of the cell cycle, and initiated tumor formation. Mechanistically, they found that CD13 protects cells from apoptosis via the ROS scavenger pathway. CD24, a mucin-like cell surface glycoprotein, was found to be a functional liver CSC marker that drives CSC genesis through STAT3-mediated NANOG regulation [10]. Xu et al. reported that DLK1+ HCC cells have characteristics similar to those of CSCs and showed higher levels of chemoresistance, colony formation, spheroid colony formation, and in vivo tumorigenicity than DLK1− cells [11]. Zhao et al. reported that α2δ1 is a functional liver CSC marker identified using a monoclonal antibody against recurrent HCC, 1B50-1, which binds to the calcium channel α2δ1 subunit. The role of α2δ1 isoform 5 in liver CSCs is related to its regulation of calcium influx through l- and N-type voltage-gated calcium channels [12]. ICAM1, which was reported as a marker of CSCs and circulating tumor cells in humans and mice, is regulated by the stem cell transcription factor NANOG [13]. Lee et al. found that CD47 is expressed in liver CSCs, which contributes to tumor initiation, self-renewal, and metastasis, and significantly affects the clinical outcome of patients. In addition, they found that CD47+ HCC cells regulate liver CSCs through the cathepsin S/protease-activated receptor 2 paracrine loop [14]. Lgr5, which is also known as a marker of liver cells following damage [35], was reported to be a potential CSC marker showing high tumorigenicity and resistance to chemotherapeutic agents [15]. Most recently, keratin19, also known as CK19, was verified as a CSC marker of HCC associated with EMT and TGFβ/SMAD signaling [16]. Using a functional approach, Muramatsu et al. identified liver CSCs by visualization system of proteasome activity and ROS level. They demonstrated that HCC subpopulation with low proteasome activity/low ROS level shows liver CSC properties and tumorigenicity in vivo. They further indicated that these liver CSCs facilitate the migration of macrophages to organize their niche, and induces metastasis by the recruitment of macrophage [57].

## Regulation of liver CSCs

### The liver CSC niche

CSCs are universally believed to reside in niches, which are specialized microenvironments that regulate adult stem cell fate by providing cues in the form of both cell-cell contacts and secreted factors. These niches maintain the principal properties of CSCs, preserve their phenotypic plasticity, protect them from the immune system, and facilitate their metastatic potential [58]. Although the liver CSC niche has not been elucidated, some evidence has suggested the potential regulation of liver CSCs by their niche. Fan et al. demonstrated that tumor-associated macrophage (TAM)-secreted TGFβ1 promotes CSC-like properties through EMT induction [59]. Wan et al. also reported that TAM-secreted IL-6 promotes the expansion of CD44+ liver CSCs and tumorigenesis [60]. Moreover, non-CSC-secreted IL-17E activates the JAK/STAT3 and NF-κB pathways in CSCs to promote their proliferation and self-renewal in HCC [61]. Lau et al. showed that cancer-associated-fibroblast-derived HGF regulates liver CSCs via the activation of FRA1 in an Erk1/2-dependent manner [62]. Lai et al. also reported that lipopolysaccharide maintains the ability of CSCs to undergo tumorigenesis, migration, invasion, and develop chemoresistance through signaling of the NF-κB/HIF1α pathway [63]. These data suggest that the liver CSC niche could be a potential therapeutic target for liver CSCs.

### Molecular signaling of liver CSCs

The Wnt/β-catenin signaling pathway plays an important role in prenatal liver development, postnatal hepatic growth, adult liver homeostasis, and liver metabolism as well as liver cancer [64]. Wnt/β-catenin activation is one of the important aberrant pathways identified in HCC. A comprehensive genome sequencing study revealed Wnt pathway-related alterations in 66% of HCCs [65]. Importantly, the activation of Wnt/β-catenin signaling has been reported in CD133 + [4], EpCAM+[8], OV6 + [7], and Lgr5 + [15] CSCs. As described above, EpCAM is a direct transcriptional target of Wnt/β-catenin signaling, and tumorigenic [53] and highly invasive EpCAM+ AFP+ HCC is regulated by Wnt/β-catenin signaling [8].

The TGFβ family plays crucial and complex physiological roles in liver cancer, which include a multitude of distinct functions, such as maintaining stem cell homeostasis, promoting fibrosis, immune modulating, as a tumor suppressor, and paradoxically, as a tumor promoter [66]. Therefore, the role of TGFβ signaling during HCC development remains controversial. The well-known role of TGFβ signaling as a tumor promoter in liver cancer is via EMT, in which epithelial cells acquire some stem cell characteristics through the activation of the SMAD3/4 pathway. Again, Fan et al. demonstrated that TAM-secreted TGFβ1 promotes CSC-like properties through EMT induction [59]. In addition, a few reports suggested that TGFβ can regulate the induction of liver CSCs. You et al. found that TGFβ1 regulates CD133+ CSCs by inhibiting the expression of the DNA methyltransferases DNMT1 and DNMT3β [67]. Conversely, the standard isoform of CD44, which is a CSC marker, regulates the TGFβ-mediated mesenchymal phenotype [48]. TGFβ is also reciprocally regulated by the TLR4/NANOG oncogenic pathway in CD133+ liver CSCs [68]. IL-6/STAT3 signaling may also maintain the population of liver CSCs in collaboration with TGFβ signaling [34, 69, 70].

The Notch signaling pathway plays a role in stem cell self-renewal and differentiation. Although the role of the Notch signaling pathway in liver cancer remains to be elucidated [71, 72], it is reportedly involved in metastasis [73] and EMT [74], which is relevant to the acquisition of a stem-like phenotype [75]. Zhu et al. reported that C8orf4 negatively regulates the self-renewal of CD13 ± CD133± liver CSCs via NOTCH2 signaling suppression [76]. The Hedgehog and HGF signaling pathways are also reportedly involved in EMT in liver cancer [77, 78]. The transcriptional coactivators TAZ and YAP, which are downstream effectors of the Hippo pathway, have oncogenic roles in human cancers. In liver cancer, Hayashi et al. demonstrated that TAZ and YAP coordinately participate in cancer progression, thereby affecting tumor growth and cancer stem-like properties [79]. ANXA3/JNK pathway has also been reported to regulate liver CSCs. Tong et al. indicated that ANXA3 promotes tumor growth and stem cell-like properties in CD133± liver CSCs through the activation of JNK pathway, moreover its neutralization suppresses HCC growth and eradicates CSC subset [80].

### Transcription factors

Reprogramming of cancer cells is an attractive concept for studying the biology of cancer stem cells. Normal somatic cells can be reprogrammed into induced pluripotent stem cells using four defined factors, that is, Oct4, Sox2, c-Myc, and Klf4 [81, 82]. Accumulating evidence suggests that these factors regulate the development and maintenance of liver CSCs. Oct4, which is also known as POU5F1, mediates the stemness of liver cancer via a positive feedback loop with the oncogene c-Jun [83]. c-Myc is a master driver of human cancers, including liver cancer, and it induces the self-renewal capacity of liver cancer cells in a p53-dependent manner [84]. Other transcription factors are also involved in the maintenance of liver CSCs. The pluripotency transcription factor NANOG is a biomarker for CSCs in HCC, and it could play an important role in the maintenance of CSC self-renewal through the IGF1R-signaling pathway [85]. Sox9 also regulates the self-renewal and tumor progression of liver CSCs through negative regulation of Numb [86].

In addition, some zinc finger transcription factors have been reported to regulate liver CSCs features. Zhu et al. demonstrated that ZIC2, which plays important roles in the early stage of organogenesis of the CNS, is highly expressed in liver CSCs and regulates the maintenance of liver CSC self-renewal through the recruitment to the NURF complex to trigger OCT4 activation [87]. SALL4, a homolog of the *Drosophila* homeotic gene spalt, is a zinc finger transcription factor expressed in embryonic stem cells that regulates their pluripotency and early embryonic development [88]. SALL4 is also expressed in fetal liver stem/progenitor cells, but not adult hepatocytes, and it plays a pivotal role in controlling the lineage commitment of liver stem/progenitor cells [89]. Recently, we and two other groups independently reported that SALL4 is a marker of a progenitor subtype of HCC, which is associated with poor prognosis, and a potential therapeutic target in HCC [90–92]. SALL4 represses its target genes, namely, phosphatase and tensin homolog and SALL1, through the epigenetic repressor NuRD complex, which contains histone deacetylases (HDACs) [93]. Indeed, high HDAC activity was detected in SALL4+ HCC cell lines, and HDAC inhibitors inhibited the proliferation of SALL4+ HCC cell lines and the expression of SALL4 gene/protein [92]. These data suggest the potential of HDAC inhibitors in the treatment of SALL4+ HCC.

### HBV/HCV, alcohol, and high-fat diet

Many cancers occur in liver that has been exposed to long-lasting inflammation induced by HBV/HCV infection, alcohol consumption, or non-alcoholic fatty liver disease [1]. Some evidence has demonstrated that these initiation factors of liver inflammation and carcinogenesis are related to the promotion of liver cancer stem properties. Arzumanyan et al. demonstrated that HBx promotes stemness factors and the development of HCC by activating β-catenin and epigenetic upregulation of miR-181 [94]. Ng et al. indicated that C-terminal- truncated HBx promotes HCC carcinogenesis through induction of CD133± liver CSCs and its tumor-initiating capacity by regulating FXR pathway and drug metabolism [95]. HCV infection of transformed human hepatocytes leads to a significant increase in the number of spheroids, the expression of EMT and CSC markers, and tumor growth in immunodeficient mice [96]. Recently, Chen and Kumar and co-workers reported that TLR4-NANOG signaling mediates the generation of liver CSCs and tumorigenesis induced by HCV infection in combination with alcohol or a high-fat diet [97, 98].

### MicroRNAs and long noncoding RNAs

MicroRNAs (miRNAs) are important key molecular components in cancer biology, and their dysregulation in liver cancer is related to CSC regulation. Wang and colleagues have explored the regulation of liver CSCs. They found that miR-181 is highly expressed in EpCAM+ AFP+ HCC cells as well as in embryonic livers and isolated hepatic stem cells, and it is functionally critical in the maintenance of EpCAM+ AFP+ HCC cells by promoting HCC stemness through targeting CDX2, GATA6, and the Wnt signaling inhibitor NLK [99]. miR-155 was also identified as a molecular target that could be used to eradicate the EpCAM+ CSC population in human HCCs [100]. miR-130b is highly expressed in CD133+ CSCs and regulates CSC self-renewal and tumorigenicity via silencing TP53INP1 [101]. miR-216a/217 and miR-125 promote EMT in HCC by inhibiting PTEN/SMAD7 and SMAD2/4, respectively [102, 103]. Most recently, Chai et al. demonstrated that miR-1246 is overexpressed in CD133± liver CSCs and likely to represent a diagnostic and prognostic biomarker for HCC. Additionally, they found that overexpression of Oct4/miR-1246 signaling axis activates Wnt/β-catenin signaling in CD133± liver CSCs by suppressing AXIN2 and GSK3β [104].

Long noncoding RNAs (lncRNAs), which are a particular class of noncoding transcripts without evident protein coding function that are reportedly involved in the regulation of stem cell differentiation, are dysregulated in human cancers [105], and are associated with the regulation of liver CSCs. It was demonstrated that lncTCF7 is highly expressed in HCC and liver CSCs, and regulates liver CSC self-renewal and tumor propagation via the activation of Wnt signaling [106]. Yuan et al. recently indicated that lncRNA-DANCR is overexpressed in HCC CSCs and correlates with poor prognosis, and it mediates increasing stemness features by interacting with β-catenin in a dependent manner by blocking miRNAs [107]. Moreover, Zhu et al. reported that lnc-β-Catm, which promotes the methylation of β-catenin, also plays a role in the maintenance of CD13 ± CD133± liver CSC self-renewal via the induction of EZH2-dependent β-catenin stabilization [108].

Thus, both miRNAs and lncRNAs play an important role in regulating the properties of liver CSCs and therefore could be therapeutic targets.

### Epigenetic alterations

Epigenetic alterations, including DNA methylation, histone modifications, polycomb repressive complex (PRC), and chromatin remodeling complex function, are mechanisms that contribute directly to carcinogenesis and CSC regulation. The relevance of epigenetic alterations in liver CSC regulation has been illustrated by some studies. Raggi et al. demonstrated that DNA methyltransferase DNMT1 inhibition-driven epigenetic reprogramming generates malignant properties and a pool of liver CSCs by long-lasting cell context-dependent memory effects [109]. The histone deacetylase SIRT1 was revealed to be necessary for the maintenance of self-renewal in liver CSCs and it transcriptionally regulated the SOX2 gene through DNA methylation-dependent epigenetic alteration [110]. By loss-of function assay using short-hairpin RNA and pharmacological inhibitor, Chiba et al. demonstrated that EZH2, a core component of PRC2, plays a role in the maintenance of liver CSCs, and therefore its inhibition is a promising therapeutic approach for eradication of liver CSCs [111]. Moreover, the chromatin remodeling factor CHD1L was associated with the malignance of HCC tumors and sustained an open-chromatin configuration at the promoter regions of two regulator genes of HCC self-renewal and differentiation [39].

### CSC-targeted therapy

Liver cancer is an aggressive tumor with a poor prognosis. The effect of current anticancer treatments, including chemotherapy, radiotherapy, and immunotherapy, is limited to the improvement of the outcome of liver cancer patients. Accumulating evidence suggests that liver CSCs are responsible for this poor prognosis because they can survive in a dominant state after treatment due to their highly resistant nature and stem cell-like abilities (self-renewal and differentiation). Therefore, the eradication of CSCs has been identified as a target to improve the outcome of liver cancer patients.

### Cell surface markers

As described above, cell surface markers, such as CD133 and EpCAM, are highly expressed on CSC populations and regulate stemness in liver CSCs. Hence, cell surface marker-targeted therapies have been proposed to eradicate liver CSCs specifically. Anti-CD133 antibody-drug conjugates inhibited CD133+ HCC growth in vitro and in vivo [112]. An anti-CD44 antibody prevented CD90+ CD44+ CSC-mediated tumor formation both locally and systemically [5]. RNAi-based EpCAM blockade decreased the EpCAM+ CSC population and inhibited both the invasion capacity and tumorigenicity of EpCAM+ cells [8]. The CD13 inhibitor ubenimex reduced the tumorigenicity and self-renewal ability of CSCs and it suppressed CD13+ tumor growth in combination with 5FU in vivo [9]. Furthermore, CD47 blockade suppressed HCC growth and increased sensitivity to chemotherapy drugs including sorafenib [14, 113]. These data further suggest that CSC marker-targeted therapy can provide a forceful synergic effect to existing chemotherapies.

### Anti-self-renewal

Since self-renewal is an important characteristic for CSC maintenance, targeting self-renewal has also been proposed for the eradication of liver CSCs. The Wnt/β-catenin signaling pathway is one of the most important pathways for self-renewal [53]. Inhibition of the Wnt/β-catenin signaling pathway by anti-miR-181 inhibitors suppressed stemness gene expression and tumorigenicity of EpCAM+ HCC [99]. In addition, the small molecular agent FH535, which is a dual inhibitor of peroxisome proliferator-activated receptor and β-catenin, also showed an inhibitory effect on the proliferation of liver CSCs [114]. Lupeol, which is a phytochemical found in fruit and vegetables, suppressed the self-renewal ability, chemoresistance, and tumorigenicity of CD133+ CSCs, and it could sensitize these cells to chemotherapeutic drugs through the PTEN-Akt-ABCG2 signaling pathway [115].

### Differentiation

Inducing the differentiation of CSCs into non-CSCs to lose their self-renewal property is another possible therapeutic approach. Oncostatin M (OSM), an IL-6-related cytokine, induces the maturation of hepatocytes. We found that the OSM receptor is expressed in the majority of EpCAM+ HCC CSCs, and OSM induces the differentiation of liver CSCs and enhances their chemosensitivity to 5-FU [116]. HNF4 is a key transcription factor for hepatocyte differentiation and the maintenance of hepatic function. HNF4α induces the differentiation of hepatoma cells into hepatocytes with a reduction of stemness gene expression and liver CSCs [117]. All-trans retinoic acid (ATRA), the carboxylic acid form of vitamin A, has an important role in the regulation of cell proliferation, differentiation, and migration during development. It has been studied widely in the prevention and treatment of many types of cancer. Zhang et al. reported that ATRA induces the differentiation of EpCAM+ HCC-CSCs, resulting in improved chemosensitivity to cisplatin [118]. BMP4, which is a signaling molecule that belongs to the TGFβ superfamily, plays a role in hepatogenesis and hepatic stem cell differentiation. Zhang et al. administered BMP4 to CD133+ HCC CSCs and found that a high-dose of exogenous BMP4 promotes their differentiation, resulting in the inhibition of CSC properties [119].

### Chemoresistance

Chemo- and radioresistance are well-recognized characteristics of CSCs; therefore, the elimination of such resistance of CSCs has been targeted with several treatment agents, as described above. Lupeol sensitized CSCs to chemotherapeutic drugs through the PTEN-Akt-ABCG2 signaling pathway [115]. CD47 blockade increased sensitivity to doxorubicin and sorafenib [14, 113]. OSM and ATRA also enhanced chemosensitivity to 5-FU and cisplatin, respectively [116, 118]. We recently reported a novel molecular target that is related to the chemoresistance of EpCAM+ liver CSCs. CHD4, a component of the NuRD complex, is recruited to UV-mediated DNA damage sites in a PARP-dependent manner [120, 121]. We found that CHD4 is highly expressed in EpCAM+ CSCs, and it plays a crucial role in the chemoresistance of these cells and the maintenance of their stemness. Furthermore, we demonstrated that targeting CHD4 using both HDAC and PARP inhibitors significantly suppressed HCC growth [122]. These results offer new mechanistic insights into the chemoresistance of HCC CSCs and suggest the clinical utility of combination therapy with HDAC/PARP inhibitors.

### Future directions

As mentioned above, many aspects of the biology of liver CSCs have been revealed through great efforts and contributions in the past decade. However, various physiological and mechanistic questions of liver CSCs still remain to be elucidated. In addition, the CSC-based diagnosis and treatment of liver cancer both need to be improved in terms of the eradication of CSCs. Liver cancer contains heterogeneous cancer cells that have multiple biomarkers, which include cell surface markers, signaling molecules, and transcription factors. Cancer treatment has generally been selected on the basis of clinical stage; therefore, it is desirable to classify cancer in detail by a combination of various biomarkers in order to provide optimal treatment to patients. Recently, newly developed technologies have provided the ability to reveal single cell heterogeneity, which has been highlighted in cancer classification, diagnosis, and treatment [123]. Circulating tumor cells (CTCs), which are also heterogeneous cells that either originate from the primary tumor or from metastatic lesions, could be used for the detection of cancer progression in peripheral blood at an early stage and for the characterization of cancer for individualized therapy. In liver cancer, Yang et al. reported that CD45− CD90+ cells are detected in the blood of 90% of liver cancer patients [6]. Furthermore, Sun et al. found that EpCAM+ CTCs are detected in 66.67% of HCC patients by CellSearch analysis and these cells displayed stem cell-like properties. They also showed that the presence of more than 2 CTCs in 7.5 mL blood is an independent prognostic factor for tumor recurrence [124]. These results suggest that studying CTC heterogeneity might also be an important approach for the investigation of cancer recurrence, prognosis, and therapeutic effects.

Treatments for the eradication of CSCs require further development to be more direct, more efficient, and more effective. One of the recent approaches to target CSCs directly is nanomedicine-based therapy, in which drug delivery and release are controlled efficiently [125]. In fact, some nanomedicine-based therapies have demonstrated efficacy against liver CSCs. Epirubicin-adsorbed nanodiamonds showed high efficacy in killing chemoresistant liver CSCs [126]. Poly lactic-co-glycolic acid-encapsulated disulfiram strongly inhibited liver CSCs, in vivo HCC growth, and metastasis in combination with copper [127].

CSC-targeted immunotherapies are also an interesting treatment strategy for eliminating liver CSCs. Among them, chimeric antigen receptor T cell (CAR-T) treatment has recently been highlighted and investigated for its clinical use in many tumors, mainly in hematopoietic tumors [128, 129]. In terms of HCC, Gao et al. developed a GPC3-targeted CAR and investigated its efficacy in vivo. They found that GPC3-targeted CAR-T suppresses HCC growth [130]. Since CSCs express single or multiple specific cell surface markers, CSC antigen-targeted CAR-T could be used for the direct eradication of CSCs. For example, EpCAM-specific CAR-expressing human peripheral blood lymphocytes inhibited tumor growth in an EpCAM+ prostate cancer metastasis mouse model [131]. In sum, although liver CSC-targeted CAR-T therapy has not been reported, it can be considered a promising approach.

While these newly developed therapeutic approaches are exceedingly attractive, their adverse effects on normal stem cells should be carefully considered because CSCs share similar features with normal stem cells, including activated markers and signaling pathways. The eradication of normal stem cells as well as CSCs would prove fatal to liver cancer patients with chronic liver disease; therefore, the future challenge is to identify specific CSC markers and develop a specific treatment for liver CSCs.

## Conclusions

Since the presence of CSCs has been recognized as one of the risk factors for a high recurrence rate and chemoresistance of liver cancers, the novel therapeutic approaches are clearly required to eradicate liver CSCs. On the basis of the current understanding of the biology of liver CSCs presented here, further efforts should be made for the application of the CSCs biology in the clinical setting to eradicate liver cancers.

## Abbreviations

ATRA: All-trans retinoic acid
CAR-T: Chimeric antigen receptor T cell
CSC: Cancer stem cell
CTC: Circulating tumor cell
EMT: Epithelial-mesenchymal transition
HBV: Hepatitis B virus
HCC: Hepatocellular carcinoma
HCV: Hepatitis C virus
HDAC: Histone deacetylase
ICC: Intrahepatic cholangiocarcinoma
IL: Interleukin
lncRNA: Long noncoding RNA
miRNA: microRNA
OSM: Oncostatin M
PRC: Polycomb repressive complex
RIP: Regulated intramembrane proteolysis
ROS: Reactive oxygen species
TAM: Tumor-associated macrophage
